# Multimodal Intraoperative Neurophysiological Monitoring in Cranial and Spinal Tumour Surgeries: A Descriptive Observational Study

**DOI:** 10.7759/cureus.49411

**Published:** 2023-11-25

**Authors:** Sangeeta Gupta, Saquib A Siddiqui, Upasna Sinha, Gaurav Gupta

**Affiliations:** 1 Physiology, All India Institute of Medical Sciences, Gorakhpur, Gorakhpur, IND; 2 Neurosurgery, Mediversal Superspeciality Hospital, Patna, IND; 3 Radiology, All India Institute of Medical Sciences, Patna, Patna, IND; 4 General Surgery, All India Institute of Medical Sciences, Gorakhpur, Gorakhpur, IND

**Keywords:** transcranial motor evoked potentials, surgeries, somatosensory evoked potentials, post-operative, neuromonitoring, neurophysiological, neurological deficits, multimodal, free-run emg, cerebellopontine angle

## Abstract

Intraoperative neurophysiological monitoring (IONM) involves monitoring the functional integrity of critical brain regions and pathways as well as identifying and preserving functionally viable neural tissues (mapping) during surgery using electrophysiological techniques. Multimodality combines various neurophysiological techniques to optimise diagnostic effectiveness and to improve the outcomes of the surgeries. The present study is a case series with comprehensive and illustrative descriptions of the neurophysiological changes in five neuromonitored cases of cranial and spinal cord tumour surgeries conducted with a multimodal approach. The cases were monitored with somatosensory evoked potentials (SSEP), transcranial motor evoked potentials (TcMEP), and both free run and triggered electromyography (fEMG and tEMG). No false negative outcomes were identified in the cases studied as there was an association of absence of change in SSEP and TcMEP both, with no neurological deficit postoperatively. Two cases were identified as having true positive neuromonitoring alerts. No false positive alerts were found in any case. Multimodal monitoring using SSEP, TcMEP, and EMG (fEMG and tEMG) in cranial and spinal tumour surgeries can improve performance with fewer false-negative and false-positive results. Neuromonitoring approaches used in combination can provide reliable information regarding postoperative neurological outcomes.

## Introduction

The delicate and elaborate organization of the brain and spinal cord contribute to the challenging nature of neurosurgical operations. Spine and cranial surgeries are often associated with substantial rates of postoperative complications; neurological deficits constitute a considerable proportion of these complications [1]. In the majority of neurosurgical procedures, the functional and normal brain will inevitably suffer damage while the abnormal tissues are being dissected or removed. These injuries could result from several techniques including cortical incisions, retraction of brain lobes or hemispheres, intraoperative bleeding and thermal injury due to electrocoagulation [2]. Postoperative neurological morbidity validates the importance of functional evaluation of the nervous system during surgical procedures. Curtailing perioperative risks and ensuring efficient monitoring may allow for the expansion of more aggressive surgical interventions, making more patients eligible for treatment. The intraoperative neuromonitoring (IONM) technique has become a vital component in many neurosurgeries. Although IONM has a long history, its application has only recently become widespread [3]. This technique primarily aims to prevent iatrogenic injuries and surgical insults to the nervous system and thus prevent secondary or postoperative neurological impairments. By conducting a continuous evaluation of the functional status of the nervous system in an anaesthetized patient during surgery, this procedure provides the surgical team with real-time information about the patient’s nervous system during an ongoing surgical manipulation. It has the potential to provide real-time feedback on critical neurological regions and pathways to the surgeon. In select circumstances, this feedback may prevent or mitigate neurological injury. As evidenced by a large multicenter study, spinal operations for deformity correction that incorporate the feedback of an experienced neurophysiology team can have as much as a 50% lower rate of neurological deficits [4].

A multimodal assessment helps in the evaluation of the functional status of sensory and motor pathways intra-operatively. Application of intraoperative neurophysiological monitoring via assessment of somatosensory evoked potential (SSEP), transcranial motor evoked potential (TcMEP) and electromyography of relevant nerve root myotomes to detect impending neurological injury has attained significance during the recent decades [5,6]. In spinal tumour surgeries, multimodal monitoring using SSEP and TcMEP has been reported to prevent surgically induced neurological complications [7]. The advantage of multimodal intraoperative neurophysiological monitoring (MIONM) is that it can make up for the shortcomings of each individual technique. It also appears to be reliable and useful for identifying perioperative neurological damage after spine surgery. The effectiveness of MIONM in spinal cord monitoring during spinal tumour resections is strongly supported by the literature [8,9]. Cerebellopontine (CP) angle tumour excision is a surgery which carries a considerable risk of facial nerve damage compared to other otological or parotid gland procedures. Facial nerve palsy is a devastating complication following CP angle tumour resections; this leads to functional as well as aesthetic deficits that can impair the patient's quality of life. In CP tumour surgeries, the technique provides the benefit of making information available to the surgical team regarding dynamic changes in the monitored structures and the possibility of cranial nerve localization using stimulation in the operating area. A previous similar study reports TcMEP along with EMG recordings as a useful method for monitoring the facial nerve during CP angle tumour surgeries [10].

The present study aims at evaluating neurophysiological changes that occurred during five different neuromonitored surgeries performed with a multimodality approach at our institution, along with a prospective evaluation for the development of new postoperative neurological deficits.

## Materials and methods

**Figure 1:**
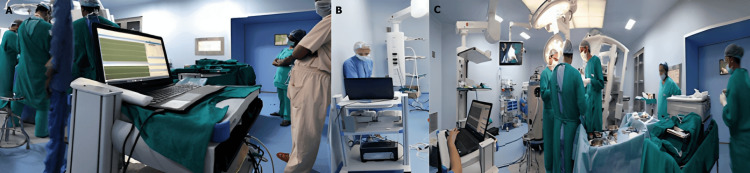
Operating room and intraoperative neurophysiological monitoring (IONM) set-up for the cases performed.

The neuromonitored surgeries were conducted in the operating room (neurosurgery and orthopaedic OTs) at All India Institute of Medical Sciences (AIIMS), Patna. Intraoperative neurophysiologic monitoring of each patient was performed on a 32-channel Xltek Protektor32 IOM system running EPWorks Software (Natus Neuro, Ontario, Canada) (Figure 1). The use of the IONM multimodalities to be tested was decided according to the location of the tumour in each patient. For SSEPs, the segmental level of the lesion determined the selection of the nerve to be stimulated. Median SSEPs were performed in order to monitor spinal cord surgery above the C6 level, ulnar nerve SSEPs for those involving the lower cervical segments (above C8) and for the surgeries below the C8 segment, posterior tibial SSEPs were performed. Upper-limb MEPs were recorded for monitoring the neuraxis when the region at risk was above the lower cervical spinal cord. When the thoracic and lumbar spinal cord were at risk, myogenic MEP recording sites included leg muscles.

Perioperative interventions

Anaesthesia was standardized according to local protocols. The total intravenous anaesthesia (TIVA) protocol was followed for all the patients and the use of volatile anaesthetic agents that could inﬂuence electrophysiologic signals was avoided by the anesthesiologist. Neuromuscular muscular blocking agents were used only at the time of induction. Intraoperative blood pressure was recorded and values below 90 mmHg (mean arterial pressure) were noted and corrected (as TcMEP amplitudes are influenced by hypotension) [11].

During surgery, several corrective measures were applied when a significant (>50%) decrease in IONM signals was registered. The surgeon also irrigated the surgical site with warm saline and temporarily stopped resection. In cases when signals did not improve, the surgeon chose whether or not to continue resection, in order to prevent iatrogenic damage. Where there was no clearly identifiable attachment plane between the tumour and the spinal cord, the surgeon stopped the intervention. When signal decreases randomly occurred and was not obviously caused by manipulation of the spinal cord, resection was usually continued.

The electrophysiologic changes during the surgeries were recorded.

Procedure

I. Intraoperative TcMEP

Subdermal needle electrodes were placed at C3 and C4 as regions for transcranial electrical stimulation (international 10-20 system) in the limb muscles; then, 9-stimuli-train with 0.05 ms duration and intra-train repetition rates of 500 Hz were delivered. The recording was performed without averaging. Filter settings were 10-1000 Hz. Sweep time and sensitivity setting were 15 ms/division and 300 microvolts/division respectively. The sweep time and amplitude settings were altered if artefact suppression was required.

As the amplitude criterion is more accepted, the measurement of TcMEP amplitudes was performed. The amplitudes were measured between the most positive and most negative points of the waveform. The surgical team was notified when myogenic MEPs decreased by a threshold percentage larger than 50% [12]. 

II. Intraoperative SSEPs

Intraoperative SSEPs were recorded from the median nerve for the upper extremity and from the posterior tibial nerve for the lower extremity. Subdermal needle electrodes were used for stimulation and recording purposes. Recording electrodes were placed at CP3 and CP4 (median SSEP) and CZ and FZ (tibial SSEP). Filter settings were 30-1000 Hz. Averaging was performed to record the responses. A 50% drop in amplitude and a 10% increase in latency was considered as a significant change in SSEP recordings [13].

III. Free-run EMG (fEMG) and Triggered EMG

Continuous free muscle electromyographic recording was performed using subdermal needle electrodes. Filter settings were 30 Hz-3000 Hz. Sensitivity and sweep time settings were 100 microvolts and 200 ms/division respectively. Sustained neurotonic discharges were considered alarm criteria in free-run EMG.

Responses to stimulation using a monopolar stimulator during the surgery were monitored (triggered EMG). Filter settings were 10 Hz-1000 Hz. Amplitude and sweep time were 700 microvolts and 7 ms/division respectively. An elevated threshold for stimulation to record compound muscle action potential (CMAP) was considered an alarm criterion.

The warning criteria in TcMEP monitoring was >50% irreversible attenuation of the waveform, while in the case of SSEPs, 50% amplitude attenuation and/or prolongation of latency >10% was regarded as significant. Sustained neurotonic discharges were considered alarm criteria in free-run EMG. An elevated threshold of stimulation (≥2 mA) to elicit a CMAP during CP angle tumour surgery was considered an alarm criterion for triggered EMG. If significant neurophysiological changes were observed during the surgeries, the anaesthesiologist was directed to check and correct the mean arterial blood pressure. Temperature and oxygen saturation were also checked. The technical integrity was ensured. If the amplitude still did not improve, even after reversal interventions, cessation of the procedure was considered. Postoperative neurological evaluations of the patients were performed first on Day 1 and then after one month. Any decline of ≥1 motor power grade (MRC scale) in cases with spinal tumour surgeries and a decline of ≥1 scale in the House-Brackmann grading system in those with CP angle tumour surgeries were defined as new-onset postoperative neurological deficits.

## Results

We have reported five neuromonitored cases of cranial and spinal tumour surgeries performed at our institute with a multimodal approach. TcMEP, SSEP, free-run and triggered EMG were the neuromonitoring modalities performed during the surgeries. 

Case 1: Vestibular schwannoma in left CP angle cistern

A 20-year-old male patient presented with weakness in the left half of the body, including the face, for the last eight months. He also complained of diminished hearing in his left ear and diminished vision in his left eye. On examination, the Glasgow Coma Scale (GCS) was E4V5M6. Cranial nerve examination revealed decreased sensation in the left half of the face and diminished jaw jerk as well as weakness of facial muscles on the left half (House-Brackmann grade II). The audiological evaluation found severe sensorineural hearing loss >80 dB nHL in the left ear with no hearing deficit in the right. 

Radiological Examination

**Figure 2:**
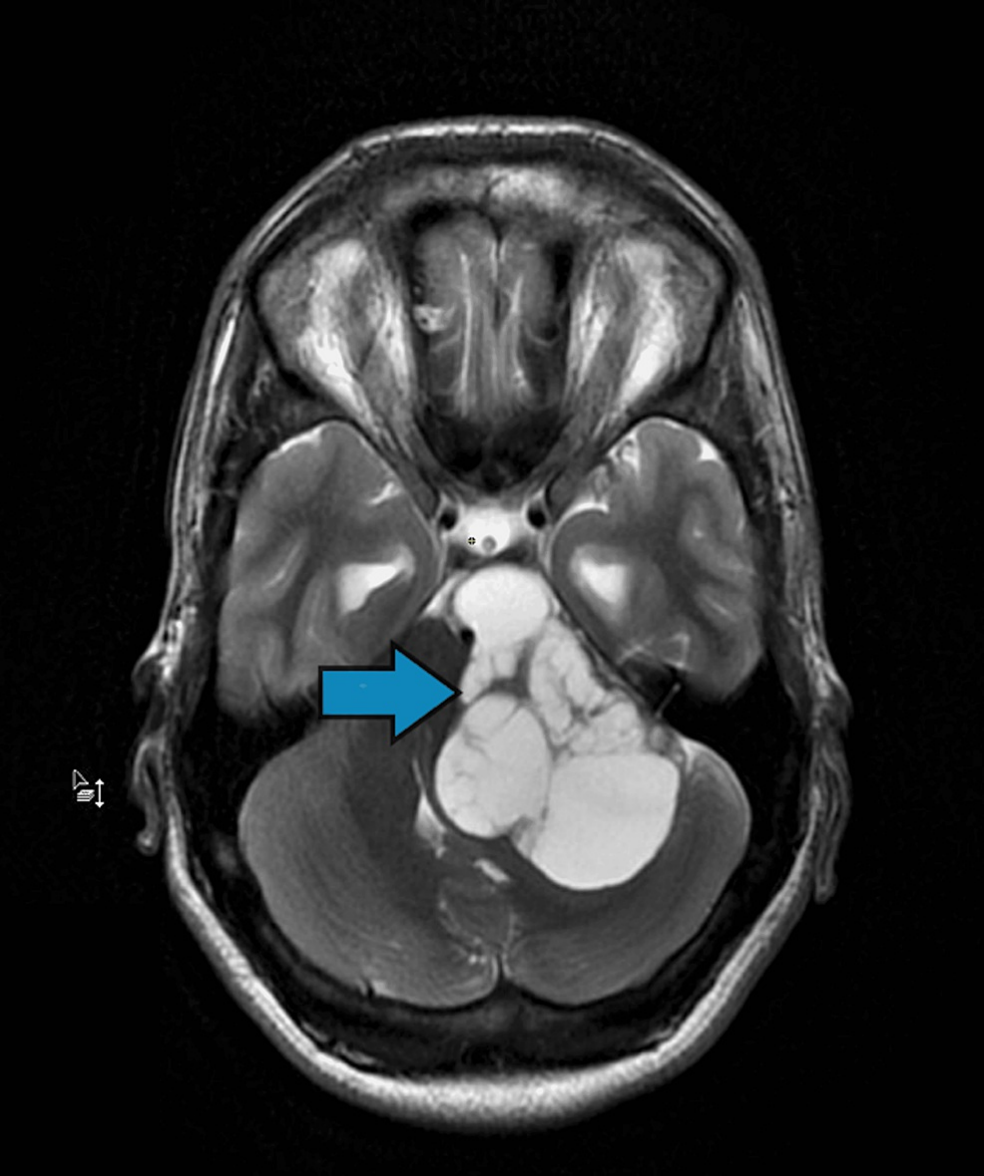
T2-weighted MRI findings showed a large lobulated cystic mass in the left CP cistern (size: 6.4 cm AP diameter and 3.97 TR) (blue arrow). Compression of the brainstem, vermis and ipsilateral cerebellar hemisphere was found. MRI: magnetic resonance imaging; CP: cerebellopontine; AP anteroposterior; TR: transverse; IAC: internal auditory canal

**Figure 3:**
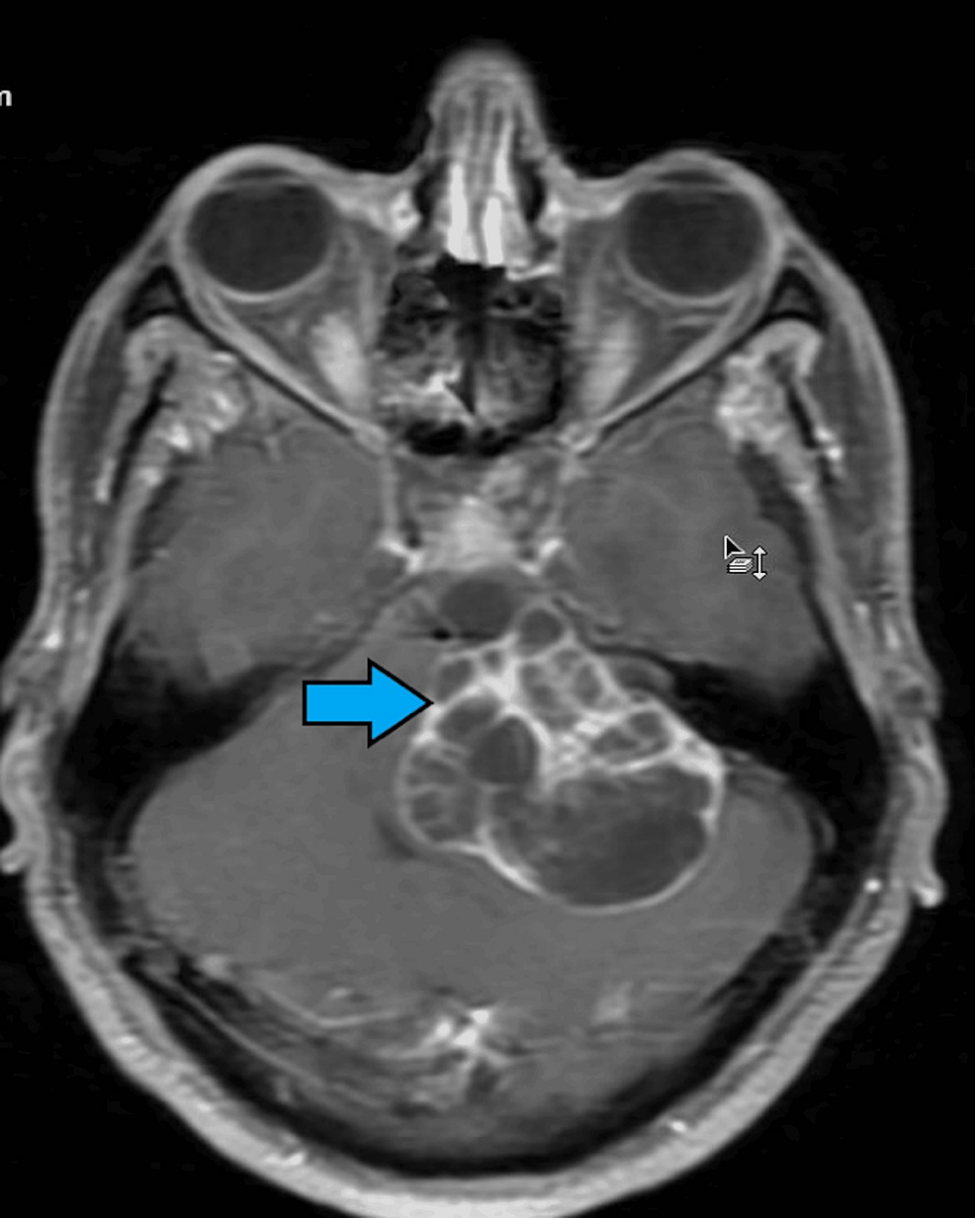
Post-contrast MRI displays the septae and wall showing moderate enhancement. The mass extends into the IAC and has an ice-cream cone appearance (blue arrow). MRI: magnetic resonance imaging; IAC: internal auditory canal

T2-weighted MRI and post-contrast MRI confirmed the presence of a large lobulated cystic mass in the left CP cistern extending into the internal auditory canal (Figures 2-3).

The diagnosis was confirmed as vestibular schwannoma in the left cerebellopontine angle cistern. The patient was planned for craniotomy and tumour excision with intraoperative neuromonitoring for the surgery.

TcMEP, triggered and free-run EMG was performed during the surgery. Brainstem auditory evoked potential (BAEP) was not conducted owing to the fact that a severe hearing deficit was already present. The TIVA protocol was followed.

Intraoperative Neurophysiologic Monitoring Findings

Facial and trigeminal nerve monitoring by triggered EMG: Needle electrodes were placed subdermally at the orbicularis oris, masseter, mentalis and orbicularis oculi muscles. Sensitivity and time base settings were 700 µv/div and 7 ms/div respectively, and a band-pass filter setting of 10-1000 Hz was utilized. A handheld monopolar stimulator was used for the stimulation.

**Figure 4:**
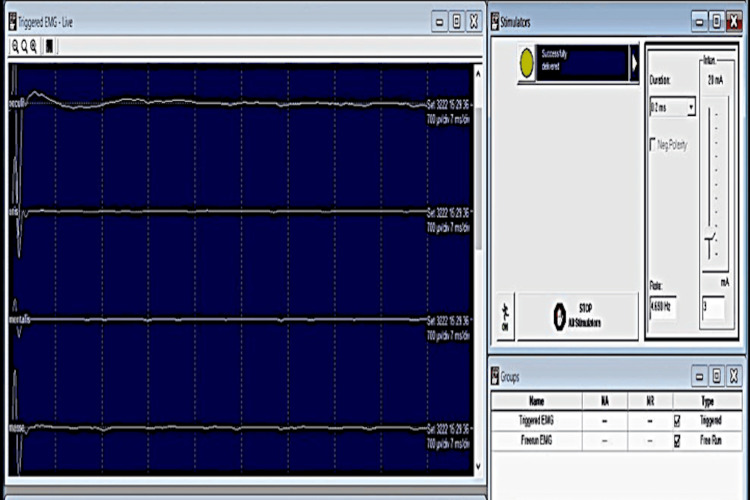
Triggered EMG response in Case 1 (Vestibular schwannoma in left CP angle cistern) The record depicts no CMAPs at a site, with the initial searching stimulus (3 mA). Sensitivity: 700 µv/div, time base settings: 7 ms/div, filter: 10-1000 Hz EMG: electromyography; CMAP: compound muscle action potentials; µv: microvolts; div: division; ms: milliseconds; Hz: Hertz

**Figure 5:**
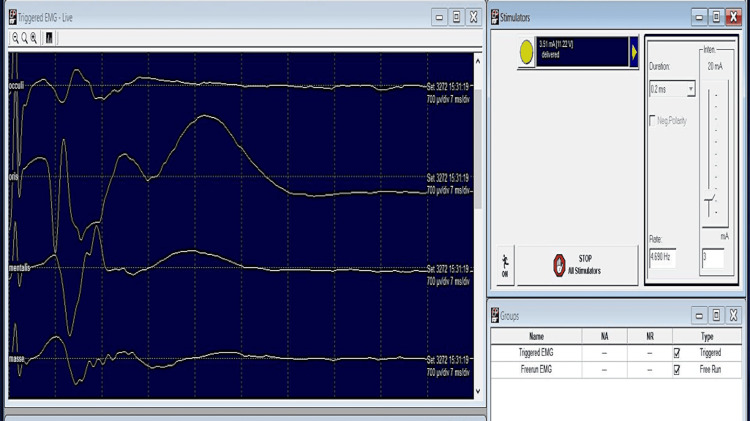
Triggered EMG record in Case 1 (vestibular schwannoma in left CP angle cistern) with initial searching stimulus (3 mA) at a distinct site, with recordable CMAPs for all the muscles tested. Sensitivity: 700 µv/div, time base settings: 7 ms/div, filter: 10-1000 Hz EMG: electromyography; mA: milliampere; CMAP: compound muscle action potentials; µv: microvolts; div: division; ms: milliseconds; Hz: Hertz

**Figure 6:**
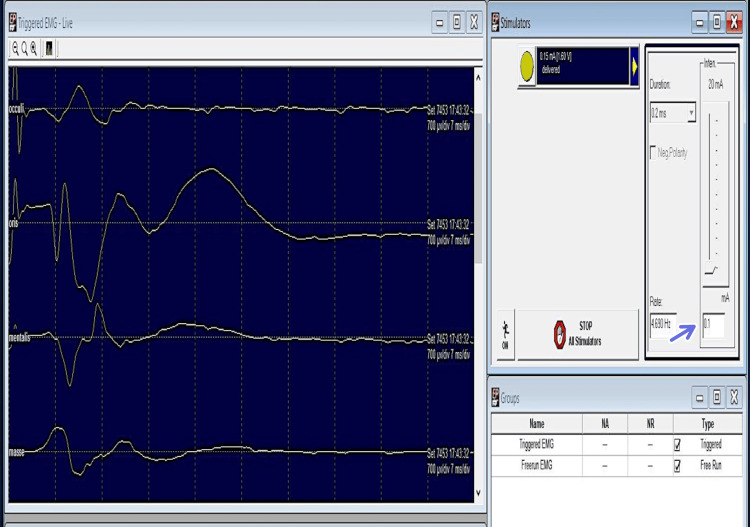
Triggered EMG record in case-1 with 0.1 mA as the threshold stimulus obtained (blue arrow). Sensitivity: 700 µv/div, time base settings: 7 ms/div, Filter: 10-1000 Hz EMG: electromyography; mA: milliampere; µv: microvolts; div: division; ms: milliseconds; Hz: Hertz

An initial searching stimulus (3 mA) was applied on the tumour surface to find the response before resection (Figure 4). When CMAPs were obtained, the stimulus strength was reduced in order to reach the threshold (Figures 5-6). In this case, a stimulus intensity of 0.1 mA (threshold stimulus) could elicit CMAP of amplitudes (600-700 µv). The facial nerve's approximate distribution pattern was traced during the procedure using free-running and triggered EMG to ensure that the nerve would be unaffected during the resection. No elevation of the threshold to record CMAP was observed during and after the resection.

TcMEP: TcMEP for facial and trigeminal nerve was recorded. Stimulation sites were C3 and C4. Five stimuli-train with 0.05 ms duration and intra-train repetition rates of 500 Hz were stimulus settings. Filter settings were 10-1000 Hz. Sweep time and sensitivity were 300 µv and 15 ms/div (altered when artefact suppression was needed). 

**Figure 7:**
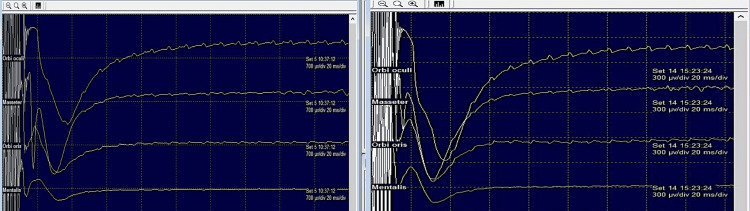
TcMEP responses seen in Case 1 for facial and trigeminal nerves. The left panel shows the baseline of TcMEP responses before the commencement of surgery and the right panel shows the same at the end of the surgery (right). TcMEP: transcranial motor evoked potential

TcMEP recording began prior to skin incision and continued until wound closure and was performed every 20-30 minutes during surgery and upon the operating surgeon’s request. No significant reduction in the amplitudes nor a decrease in the duration or alteration in the complexity of the waveforms was found (Figure 7). 

Postoperative Clinical Examination

The patient was found with no new facial nerve deficits during the follow-up examination on Day 1 and after one month postoperatively (Table 1).

**Table 1:** Tumour location, intraoperative neuromonitoring modalities, neurophysiologic changes and postoperative findings in the patients TcMEP: transcranial motor-evoked potentials; EMG: electromyography; fEMG: free-run electromyography; tEMG: triggered electromyography; SSEP: somatosensory evoked potentials; CMAP: compound muscle action potential

Case no	Age	Sex	Tumour and location	Modalities performed	Neurophysiologic changes observed during surgery	Pre-operative neurologic deficit	Postoperative neurologic deficit
1	20	Male	Vestibular schwannoma in left cerebellopontine angle cistern	TcMEP, fEMG and tEMG	No elevated threshold in tEMG. No significant reduction in TcMEP amplitudes.	House-Brackmann grade II	House-Brackmann grade II on Day 1 and after 1 month). No new facial nerve deficits)
2	40	Male	Vestibular schwannoma in left cerebellopontine angle cistern	TcMEP, fEMG and tEMG	Persistent elevation of threshold in tEMG. Severe attenuation in TcMEP amplitudes.	House-Brackmann grade II	House-Brackmann grade VI after 1 month
3	18	Male	Large giant cell tumour invading sacrum as well as iliac bones	TcMEP, fEMG and tEMG	No increase in threshold in tEMG, no reduction in TcMEP amplitudes from baseline levels	Motor power: grade 3; bladder and bowel continence: abnormal	Motor power grade 3 at Day 1 and grade 4 after 1 month). Bladder and bowel continence: Normal (after 1 month)
4	35	Female	Intramedullary ependymoma (C7-D4)	TcMEP, fEMG and tEMG, SSEP	Temporary reduction of TcMEP CMAP (68% attenuation). No other significant alerts	Motor power (right lower limb): grade 1	Motor power (right lower limb) after 1 month: grade 4
5	14	Male	Intramedullary dermoid (D12-L1) spinal cord	TcMEP, fEMG and tEMG, SSEP	No significant alerts	Motor power: grade 5	Motor power: grade 5 (Day 1 and after 1 month) No new neurological deficit.

Case 2: vestibular schwannoma in left CP angle cistern

A 40-year-old male complained of ataxia and hearing loss in his left ear for four months. His House-Brackmann score was grade II, indicating a slight weakness. Audiological examination revealed a severe sensorineural hearing loss in the left ear with no hearing deficit in the right (pure tone audiometry).

Radiological examination revealed a large well-defined left CP angle mass (size: 2.6 cm AP and 3.6 cm TR diameter) with extension through left IAC along with mild obstructive hydrocephalus. The diagnosis was confirmed as vestibular schwannoma in the left cerebellopontine angle cistern. TcMEP, triggered and free-run EMG were performed during the surgeries. BAEP was not conducted as the affected side had a severe hearing deficit at the time of presentation. TIVA protocol was followed.

Intraoperative Neurophysiologic Monitoring Findings

**Figure 8:**
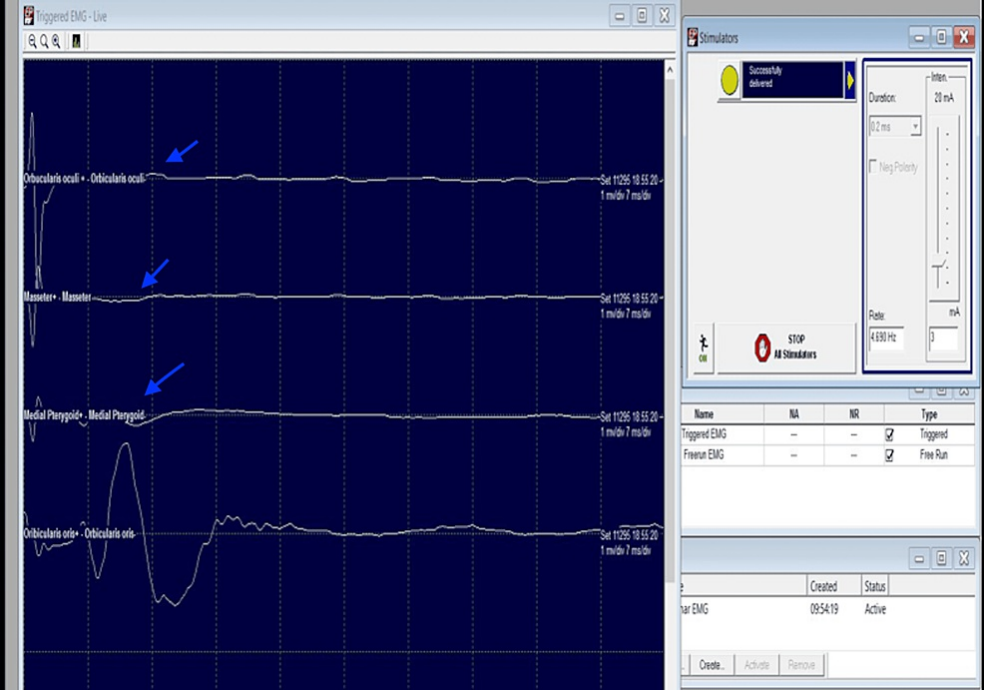
Triggered EMG record in Case 2 (vestibular schwannoma in left CP angle) with elevated threshold stimulus (3 mA) and no recordable CMAPs (blue arrows) except for orbicularis oris muscle. Sensitivity: 700 µv/div, time base settings: 7 ms/div, Filter: 10-1000 Hz. EMG: electromyography; mA: milliampere; CMAP: compound muscle action potentials; µv: microvolts; div: division; ms: milliseconds; Hz: Hertz.

The threshold of the stimulus to elicit CMAP in facial muscles was found to be 1 mA in triggered EMG. After about 1 hour of surgery, no response could be obtained at a similar threshold. After another hour, the threshold was 3 mA, with response only in one muscle (orbicularis oris). This elevation of threshold stimulus (3 mA) remained consistently elevated till the wound closure (Figure 8). TcMEP amplitudes showed severe attenuation of waveforms concurrent with the EMG findings. Neurophysiologic changes did not improve even after prompt reversal interventions were performed.

Postoperative Clinical Examination

The House-Brackmann score was grade VI one month postoperatively.

Case 3: A rare case of a large sacral giant cell tumour

**Figure 9:**
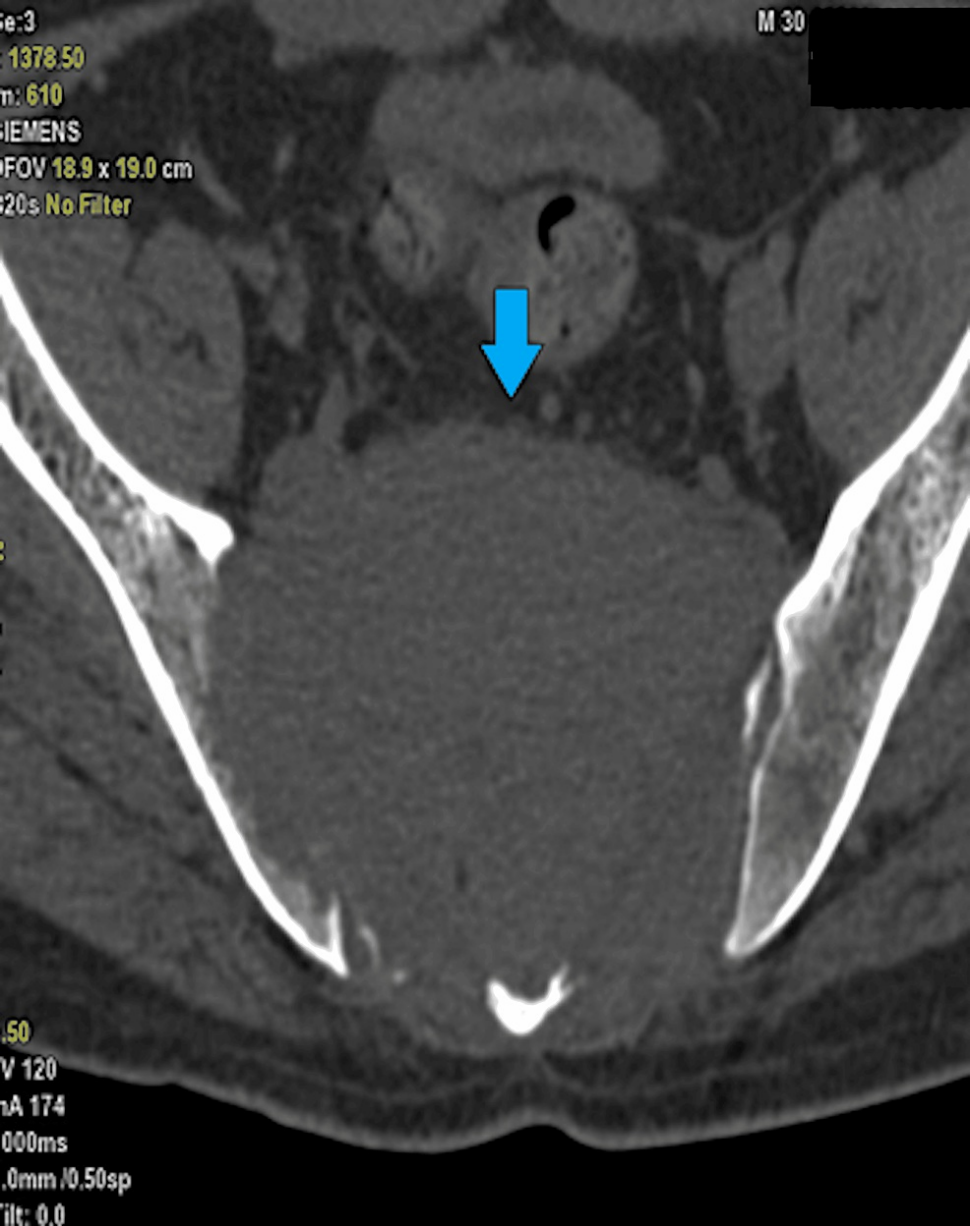
CT imaging in axial view showing a large giant cell tumour invading the sacrum and iliac bones (blue arrow). CT: computed tomography

**Figure 10:**
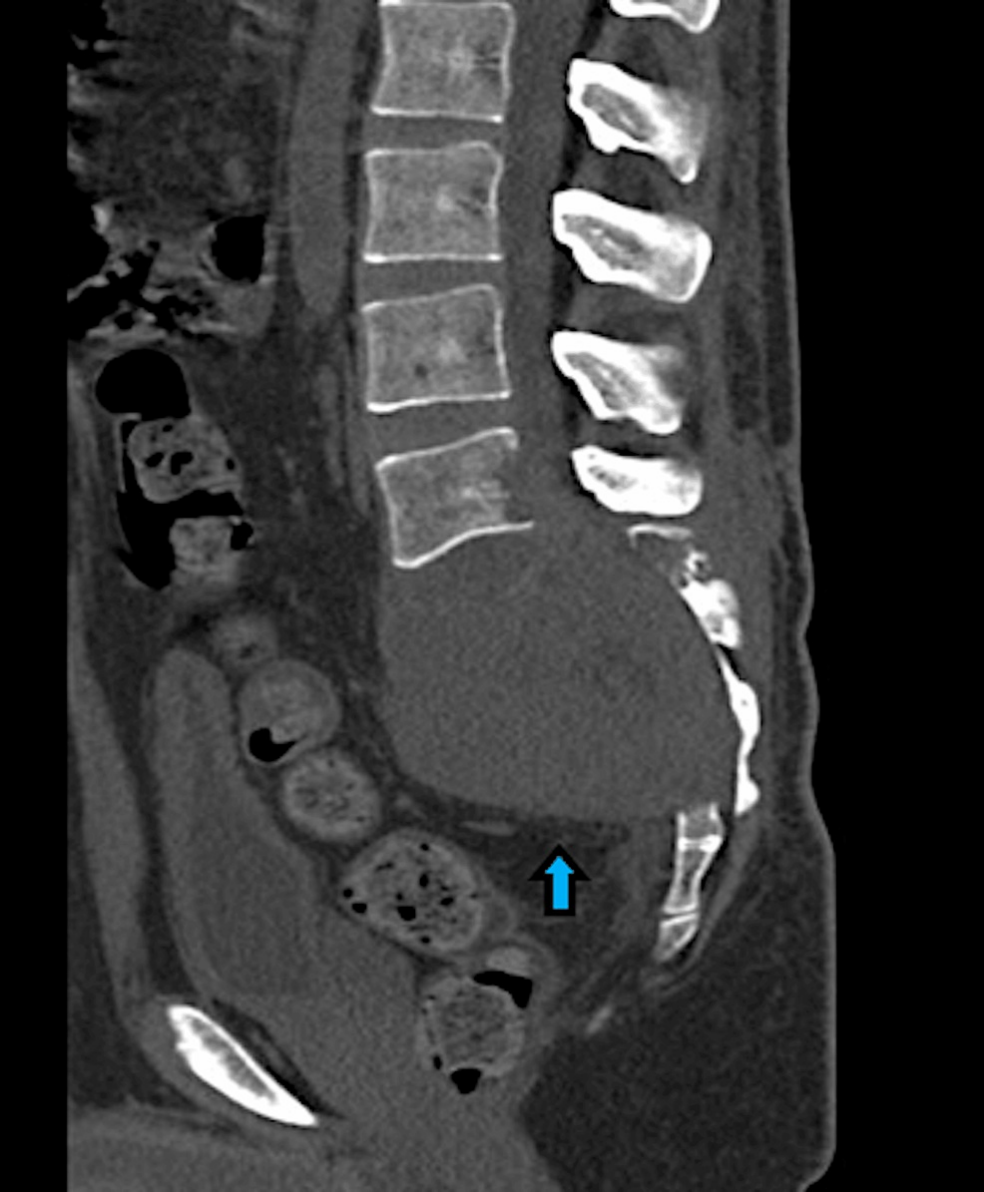
CT imaging in sagittal view showing a large giant cell tumour invading the sacrum and iliac bones (blue arrow). CT: computed tomography

An 18-year-old male presented with weakness in the lower limbs, incontinent bladder and bowel, and pain in the back and buttocks. On examination, his lower limb muscles had grade 3 power (MRC [Medical Research Council] muscle power scale). Radiological examination (CT spine) suggested a large giant cell tumour invading the sacrum as well as iliac bones (Figures 9-10).

The diagnosis was confirmed to be a sacral giant cell tumour. TIVA protocol was followed and following the induction of anaesthesia, electrodes were placed to monitor the functions of the anal sphincter and lower limb muscles. External anal sphincter (S2, 3 and 4), abductor hallucis (S1 and 2) muscles and other lower limb muscles were selected for the placement of needle electrodes. The tumour was exposed, encasing the sacral nerves. During this part of the procedure, nerve monitoring becomes imperative. Gross total excision was done. Enbloc resection in such eloquent regions is usually not possible for large and extensive tumours. IONM modalities tested were TcMEP, triggered EMG and free-run EMG. 

Intraoperative Neurophysiologic Monitoring Findings

**Figure 11:**
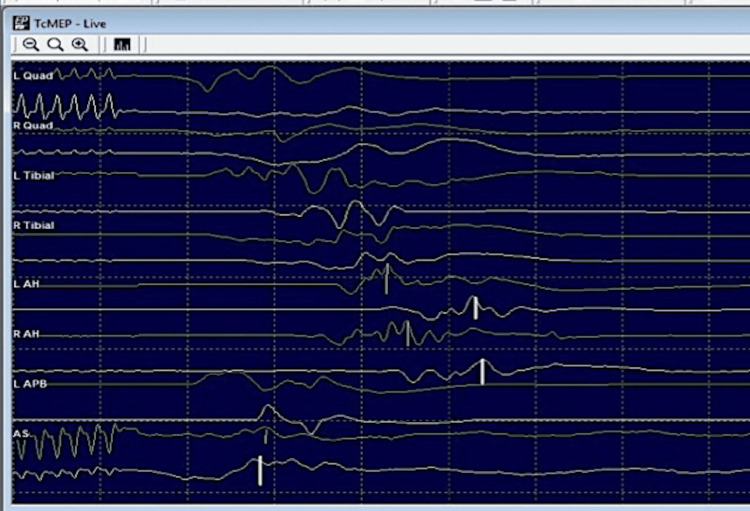
Trans-cranial motor evoked potentials (Tc-MEP) findings in a patient with sacral giant cell tumour (Case 3) near the end of the surgery compared with the baseline records (green traces). Sweep time and sensitivity: 300 µv and 10 ms/div respectively. Recorded from the quadriceps femoris, tibialis anterior, abductor hallucis and external anal sphincter µv: microvolts; div: division; ms: milliseconds

TcMEP: No significant reduction in the TcMEP amplitudes from the baseline was noted in the external anal sphincter and abductor hallucis (S1-S3) and the lower limb muscles (L2-S1) (Figure 11). 

**Figure 12:**
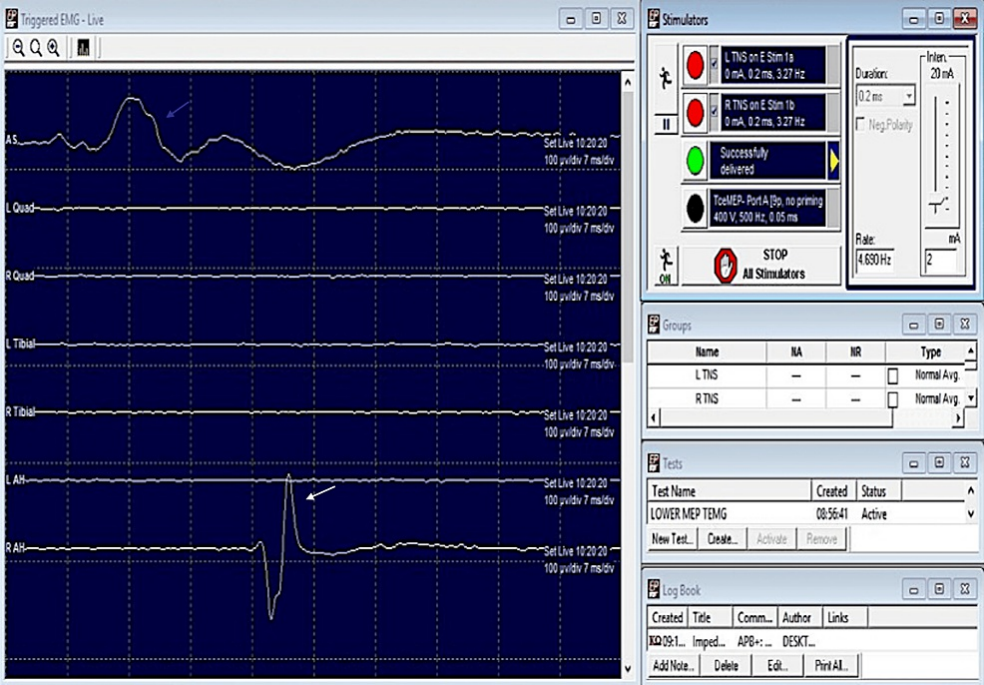
Triggered EMG findings (Case 3: sacral giant cell tumour) for EAS (blue arrow) and AH (white arrow) muscles at 2 mA (threshold stimulus for the CMAPs). EMG: electromyography; EAS: external anal sphincter; AH: abductor hallucis; mA: milliampere; CMAP: compound muscle action potentials

Triggered EMG findings: Triggered EMG response could be obtained with CMAP amplitudes of 80 microvolts and 150 microvolts at a threshold stimulus of 2 mA stimulus intensity for EAS and AH muscles respectively (Figure 12). No elevation of threshold stimulus was observed till the end of the surgery.

Postoperative Clinical Examination

Motor examination (lower limb muscles): Muscle power was grade 3 on Day 1 and achieved grade 4 muscle power after one month. Bladder and bowel continence was normal after one month (Table 1).

Case 4: Intramedullary ependymoma (C7-D4 level)

A 35-year-old female presented with bilateral weakness in the lower limbs, pain in the right upper and lower limbs along with a tingling sensation in the right upper limb. She provided a history of similar complaints two to three years prior to the presentation. On examination, the GCS was found to be 15. The muscle power grade for the right lower limb muscles was 1/5, while the rest of the muscles were found to have grade 5 power. Deep tendon reflexes for the lower limbs were exaggerated.

Radiological Examination (MRI Findings)

**Figure 13:**
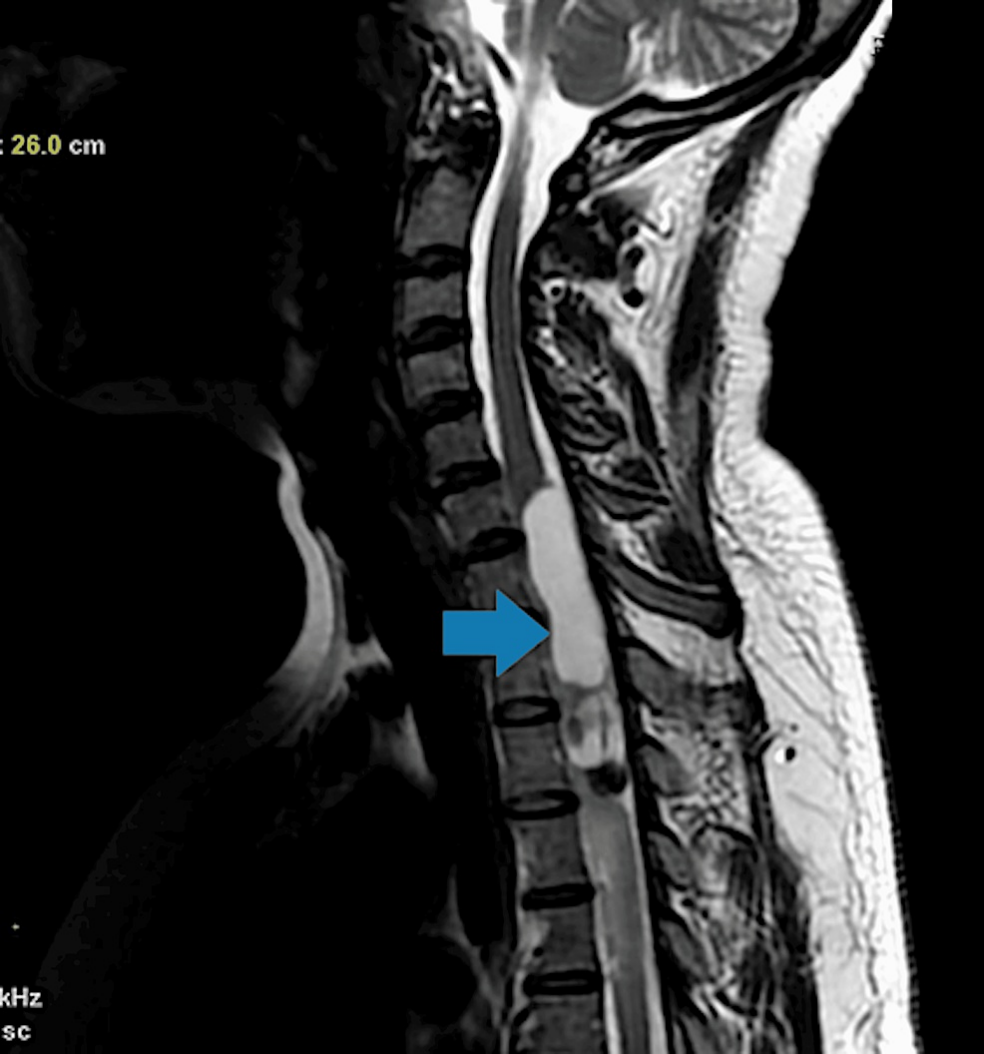
T2-weighted MRI depicting an elongated cystic lesion evident at the C7-D4 level of the spinal cord (blue arrow). MRI: magnetic resonance imaging

**Figure 14:**
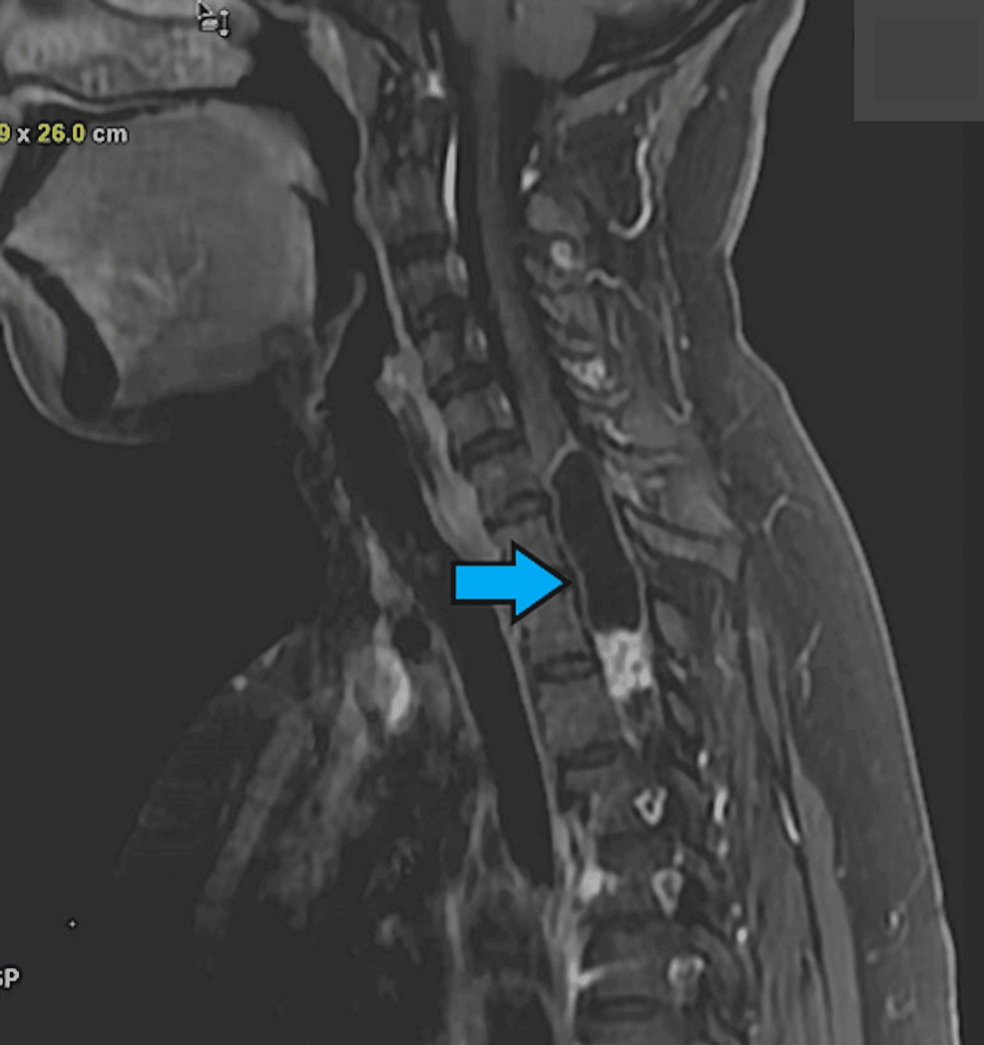
Post-contrast MRI depicting an elongated cystic lesion with a solid component at the C7-D4 level of the spinal cord (blue arrow). MRI: magnetic resonance imaging

An elongated cystic lesion with a solid component (post-contrast MRI) was evident at the C7-D4 level of the spinal cord (Figures 13-14). The case was diagnosed as intramedullary ependymoma at the C7-D4 level of the spinal cord. C7-D2 laminectomy was planned with IONM.

Intraoperative Neurophysiologic Monitoring Findings

Modalities tested were TcMEP, triggered EMG, free-run EMG and SSEP. TIVA protocol was followed. Recordings were obtained from the abductor pollicis brevis, quadriceps femoris, tibialis anterior and abductor hallucis muscles on both sides.

TcMEP: CMAPs were recordable from all the muscles tested at the baseline. However, a transient reduction of TcMEP amplitudes (68% from baseline) was observed after one hour of surgery. A fall in mean arterial pressure was detected (<90 mmHg). Correction of the hypotension restored the records and the amplitudes regained the baseline values shortly. No significant reduction in the amplitudes was found thereafter, until the wound closure (as compared to the baseline record). 

SSEP: Median nerve and tibial nerve SSEP were performed. Subdermal needle electrodes were used for recording and stimulation purposes. Recording electrodes were placed at CP3 and CP4 sites. Filter settings were 30-1000 Hz. Averaging was done to obtain the responses.

Median nerve SSEP: Median nerve SSEP at the baseline (N20 latency) were 18.2 and 17.5 ms on the left and right sides respectively. No increase in latency was found during/at the end of the surgery. No significant reduction in the amplitude was obtained.

**Figure 15:**
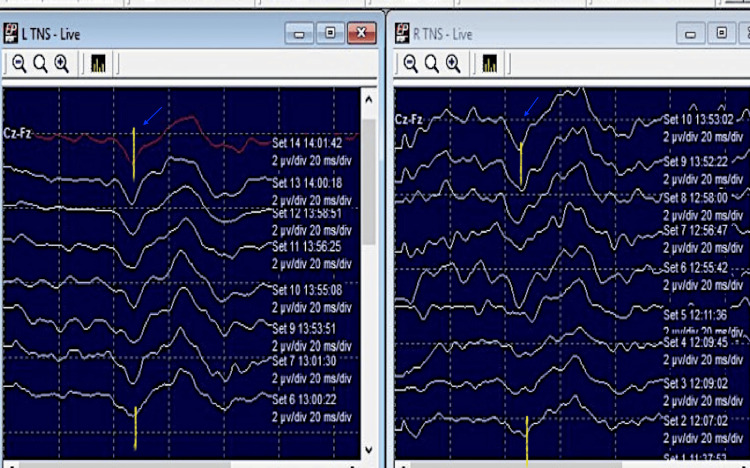
Tibial SSEP in a patient with intramedullary ependymoma (C7-D4) (case 4) with no significant increase in P37 latency (blue arrows). SSEP: somatosensory evoked potentials

Tibial nerve SSEP: Tibial nerve SSEP as compared to the baseline (P37: 47 ms [left] and 45 ms [right]) did not reveal an increase in the latency; also, there was no significant reduction in the amplitude (Figure 15).

Postoperative Clinical Examination

Motor examination after one month revealed the muscle power grade as 4 (preoperative: grade 1) (Table 1).

Case 5: Intramedullary dermoid (D12-L1) spinal cord

A 14-year-old male presented with complaints of tingling sensations in the left lower limb for two months, and slight difficulty in walking. On examination, a slight limping gait was observed. CNS examination revealed left calf muscle atrophy. Tone and power (grade 5) were normal. Nerve conduction studies were normal.

Radiological Findings

**Figure 16:**
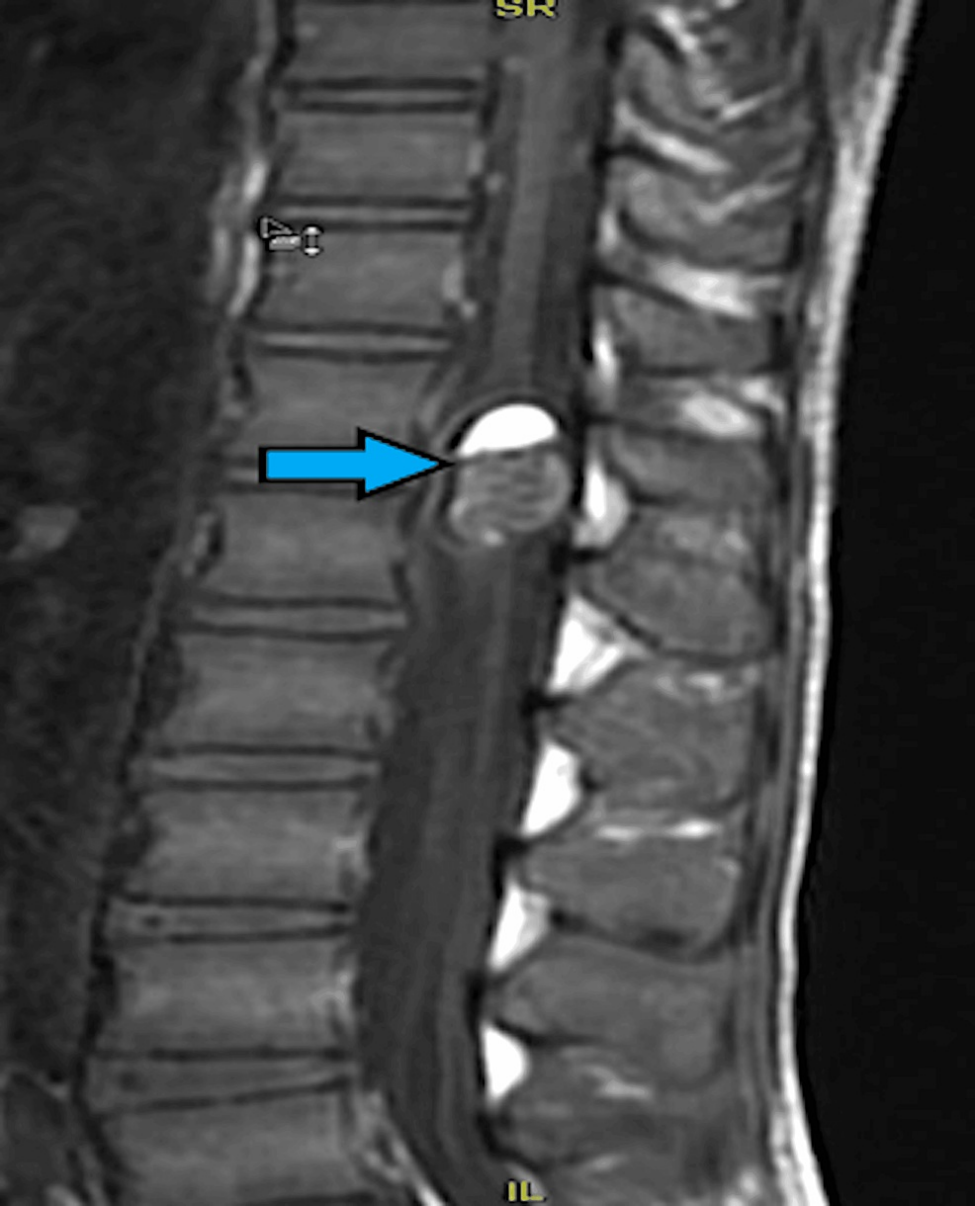
T1-weighted MRI depicts a well-defined oval intramedullary lesion within the spinal cord at D12-L1 level causing expansion (size: 1.4 cm anteroposterior and 1.85 cm transverse) more towards the left side of the cord with areas of T1 hyperintensity (blue arrow).

**Figure 17:**
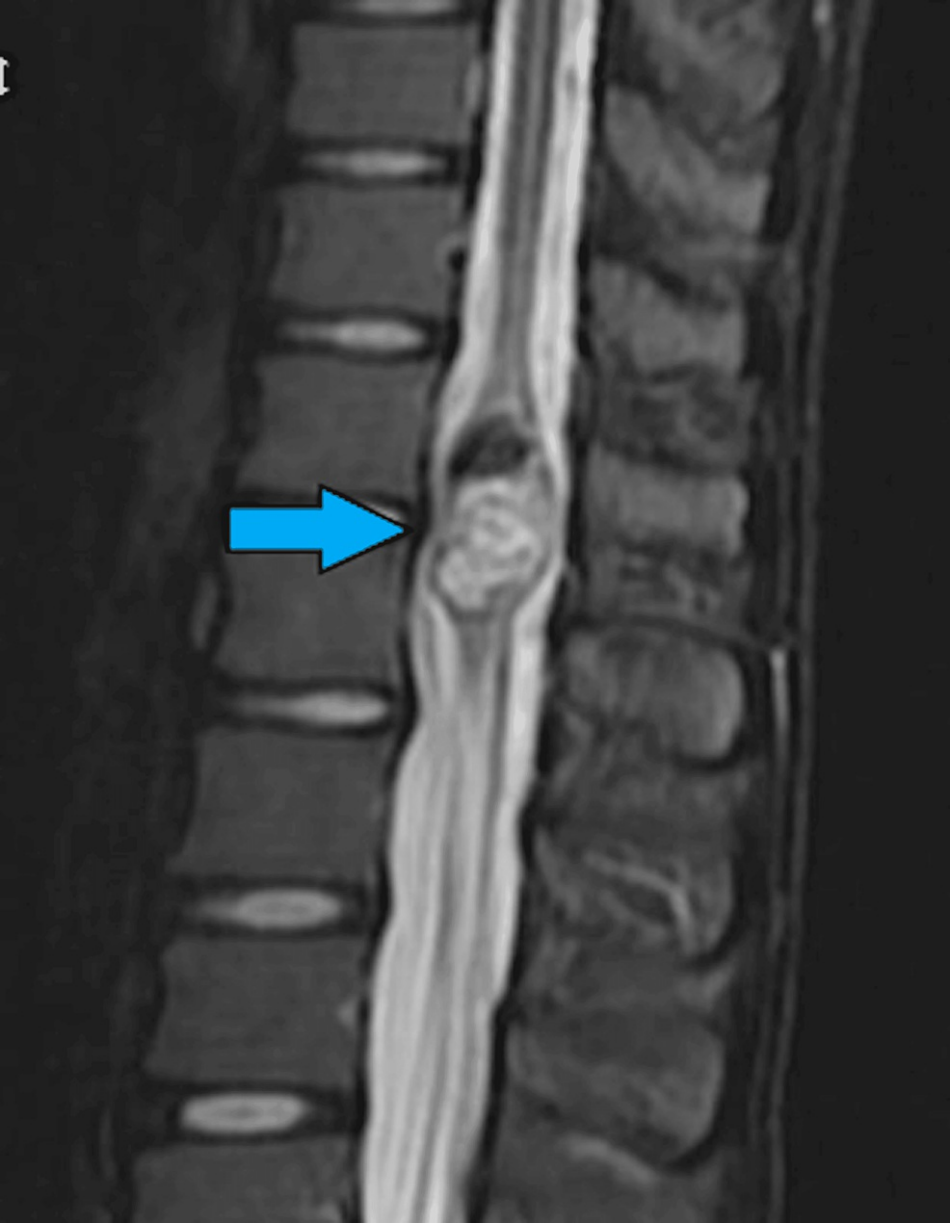
T2 fat-suppressed MRI depicting an intramedullary lesion within the spinal cord at D12-L1 level (size: 1.4 cm anteroposterior and 1.85 cm transverse; blue arrow).

MRI depicted a well-defined oval intramedullary lesion within the spinal cord at the D12-L1 level (Figures 16-17). The lesion was diagnosed as an intramedullary dermoid at the D12-L1 level of the spinal cord. A laminectomy was planned with IONM.

IONM modalities tested were TcMEP, triggered EMG, free0run EMG and SSEP. TIVA protocol was followed. Recordings were done from abductor pollicis brevis (right), quadriceps femoris, tibialis anterior and Iliopsoas muscles on both sides.

Intraoperative Neurophysiologic Monitoring Findings

**Figure 18:**
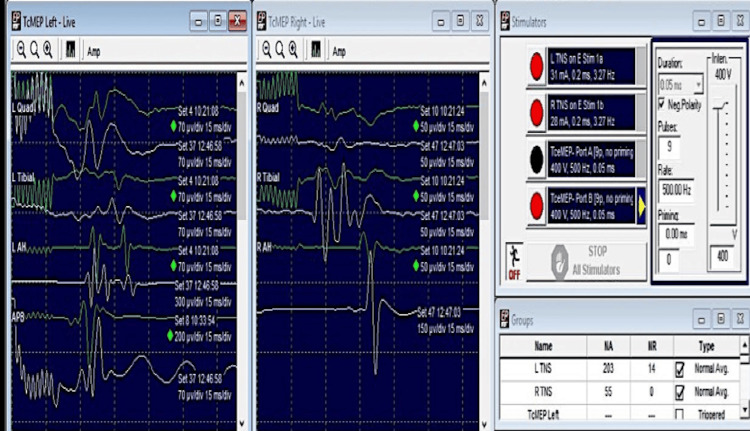
TcMEP findings compared with the baseline (green traces) in a patient with intramedullary dermoid (D12-L1) spinal cord (Case 5) TcMEP: transcranial motor evoked potentials

TcMEP: Recordable CMAPs were obtained from all the muscles at the baseline. No significant reduction in the amplitudes was found at the time of wound closure as compared to the baseline record (Figure 18). 

**Figure 19:**
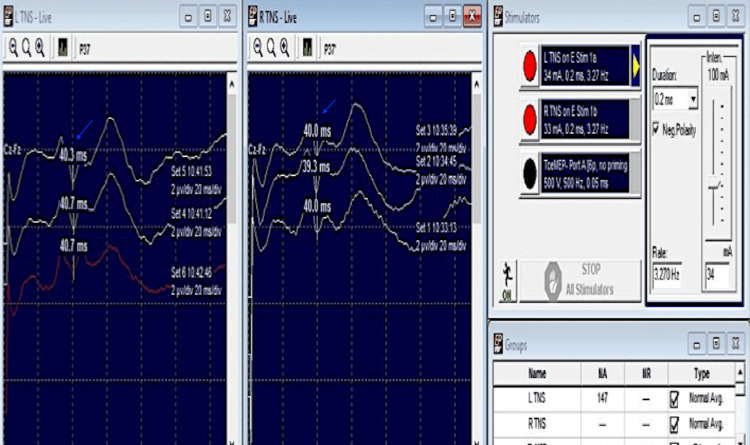
Tibial SSEP recordings in a patient with intramedullary dermoid (D12-L1) spinal cord (Case 5) at the end of the surgery with no significant alteration in P37 latencies (blue arrows). SSEP: Somatosensory evoked potentials

Tibial nerve SSEP: tibial nerve SSEP as compared to the baseline did not reveal an increase in latency (P37: 40.7 ms and 40 ms in left and right tibial SSEP at the end of the surgery) nor significant reduction in the amplitude was found (Figure 19).

Median nerve SSEP: Median nerve SSEP as compared to the baseline did not reveal any significant increase in latency (N20 were 22.5 ms and 23 ms for left and right MNS respectively at the end of the procedure) nor significant reduction in the amplitude was found.

Triggered EMG: Recordable CMAPs could be obtained from the muscles with the threshold stimulus intensities till the end of the procedure.

Postoperative Clinical Examination 

Motor examination revealed the muscle power grade as 5 at the follow-up on Day 1 and after one month postoperatively (Table 1). The patient was ambulatory.

Summary of the salient findings

In Case 1 with vestibular schwannoma in the left cerebellopontine angle, no critical changes were detected intraoperatively. The facial nerve functional status was found to be preserved postoperatively (Day 1 and one month) with no deterioration in House-Brackmann grade (grade II pre-operatively). It was, hence, a true negative neuromonitoring alert. On the other hand, Case 2 with vestibular schwannoma in the left cerebellopontine angle had significant alerts in tEMG and TcMEP with severe deterioration of House-Brackmann score at one month postoperatively. This case signified a true positive outcome.

In cases with spinal tumours (Cases 3 and 5), no temporary/permanent neurophysiological changes were observed intraoperatively. Postoperative examination revealed improved motor power grades in Case 2 (at one month), while preservation of preoperative motor power was seen in Case 4 (Table 1). These were true negative outcomes with no significant neurophysiological changes intraoperatively and no new postoperative neurological deficit.

However, in Case 4 with intramedullary ependymoma (C7-D4 level), a waveform attenuation (68%) of TcMEP CMAP was detected. The surgical procedure was stopped temporarily. Mean arterial pressure (MAP), temperature and oxygen saturation were checked. The fall in MAP was detected which was then corrected. Irrigation of the surgical ﬁeld using warm saline solution was performed. TcMEP amplitudes were found to be restored after a short period of time. No other temporary/sustained neurophysiological changes were found during the surgery. As the changes responded favourably to intervention and no postoperative neurological deficit was detected, it was a true positive alert [14,15].

## Discussion

The present study documents the results of intraoperative neuromonitoring with the use of multimodal techniques during cranial and spinal tumour surgeries. Case 1 with vestibular schwannoma in the left CP angle cistern was neuromonitored with TcMEP, free-run EMG and triggered EMG. The size of the tumour in this case was 6.4 cm, based on the maximum diameter of the extrameatal portion of the tumour. It has been well documented that facial nerve outcomes largely depend on the objective tumour size parameters [16]. Previous reports state that the proportion of cases with a good facial nerve outcome, i.e. House-Brackmann (HB) grades I and II, drops to about 50% with the larger tumours (>3.5 cm) [17]. It has been widely accepted that damage to the cranial nerve during tumour removal is unavoidable in cases with bigger tumours. However, a significant reduction in facial paralysis has been documented in previous studies with the use of intraoperative neuromonitoring techniques [18]. Moreover, a previous study emphasized the use of TcMEP in addition to continuous EMG recordings for monitoring the facial nerve during surgery. They further stated that due to the excessive sensitivity of the EMG technique with the neuromuscular blockers, the application of additional methods is required [10]. The above case had well-preserved facial nerve function postoperatively. The positive association between the absence of neurophysiologic changes and no new neurologic deficits can be largely attributed to the use of multimodal intraoperative neuromonitoring techniques during the surgery. Similarly, persistent significant alerts in triggered EMG and TcMEP signals in another case with vestibular schwannoma in the CP angle (Case 2) were indicative of irreversible injury; this was followed by a postoperative neurologic deficit. MIONM could offer a true positive outcome in this case as well.

A large giant cell tumour (GCT) of the sacrum (Case 3) often presents late, usually after massive enlargement of the tumour. The tumour's anatomical placement and near proximity to the nerve roots pose challenges for surgical excision. Bowel and bladder function may be compromised when the sacral nerve roots are not preserved. A multimodal approach in the neuromonitoring was adopted in the surgery and there was a true negative outcome in this case with improvement in muscle power grade and normal bladder bowel continence (at one month postoperatively). 

Cases with intramedullary spinal cord tumours (IMSCT) are rare, accounting for only 2-4% of central nervous system tumours and 15% of all primary intradural tumours in adults [19]. SSEPs were used alone prior to the emergence of MEP during intramedullary spinal cord tumour surgery with the belief that changes in SSEPs specifically represented spinal cord dysfunction [20]. However, it was found that false negative results occurred during surgeries monitored solely with SEPs [21]. Many studies demonstrated improved outcomes following surgery for intramedullary spinal tumours with MEP monitoring [22]. A similar study recommended the inclusion of fEMG monitoring in IOM [23]. 

In the present study, we did not observe false-negative monitoring. There was no case that showed a change in both SSEP and TcMEP and still developed neurological deficit. Also, no false positive outcome was found. The specificity and sensitivity of the application of multimodal intraoperative monitoring have been documented during different spinal surgical procedures [24-26]. Sutter et al. provided a detailed analysis of a large patient population and reported a sensitivity of 89% and specificity of 99% for MIOM [27].

Limitations of the study

A combination of TcMEP (muscle) and D-wave monitoring has been suggested to be the gold standard for IMSCT surgery. We could not use D-wave monitoring due to technical constraints. Our protocol of choice was a combination of transcranial muscle motor-evoked potentials (m-MEP), SSEP, free-run and triggered EMG, without D-wave monitoring. D-waves are elicited using the same electrode montage as that for m-MEP with recording electrodes in the epidural space. A persistent stable D-wave predicts good motor outcomes even if muscle MEPs are absent/lost during surgery. However, the invasive nature and fewer insurance indications continue to be the limitations for its use. Also, we used monopolar stimulators in triggered EMG where bipolar stimulators have been reported to be more accurate when discrete differentiation of nerve from adjacent tissue is required. We attempted to overcome this by progressively decreasing the current intensity as the nerve was approached.

The study included a follow-up duration of one month postoperatively. A longer period of follow-up could have resulted in more precise estimations of the outcomes.

## Conclusions

Multimodal monitoring employing SSEP, TcMEP, and EMG (fEMG and tEMG) in cranial and spinal tumour surgery can yield better performance with reduced false negative and false positive outcomes. It helps minimise surgically induced neurological complications and allows the surgeon to make prompt reversal interventions and alterations to the surgical technique.
